# Dr. Home

**Published:** 1845-05

**Authors:** 


*Dr. Home.*—Dr. James Home, late professor of Medicine in the Northern University, was at his death, on the 5th of December, eighty-four years of age. It is only two years since he relinquished his chair. He was a professor for forty years, having succeeded his father, Francis, in 1779. On *Materia Medica*, Dr. Home was very popular in the University, particularly during the last years of his holding that professorship. This increase of popularity was chiefly owing to his having adopted the new views of Sir Humphrey Davy on the subject of chlorine. For while Murray, of the Edinburgh School, keenly opposed those views, seeking to overthrow them by experiment, and Hope hesitated to adopt them, Home remodelled this part of his lectures, and so closely followed Davy's nomenclature and explanations, that the student did not learn from him that there ever had been any other. In the chair of Medicine, no teacher took greater pains with his lectures; yet their success latterly was far from proportioned to the pains bestowed upon them. In the clinical wards, he had the reputation of being attached perhaps a little too much to the lancet.—*Northern Jour. of Med.*

